# A Comparison of *Aggregatibacter actinomycetemcomitans (Aa)* Virulence Traits in a Rat Model for Periodontal Disease

**DOI:** 10.1371/journal.pone.0069382

**Published:** 2013-07-23

**Authors:** Helen Schreiner, Yu Li, Joshua Cline, Vincent K. Tsiagbe, Daniel H. Fine

**Affiliations:** 1 Department of Oral Biology, University of Medicine and Dentistry of New Jersey, New Jersey Dental School, Newark, New Jersey, United States of America; 2 Graduate School of Biomedical Sciences, University of Medicine and Dentistry of New Jersey, Newark, New Jersey, United States of America; 3 Department of Pathology and Laboratory Medicine, New Jersey Medical School, University of Medicine and Dentistry of New Jersey, Newark, New Jersey, United States of America; Charité-University Medicine Berlin, Germany

## Abstract

Our aim was to explore the effects of Cytolethal Distending toxin (Cdt) in a well established rat model of periodontal disease where leukotoxin (LtxA) was thought to have no known effect. *In vitro* studies, were used to assess CdtB activity using *Aa* Leukotoxin as a negative control. These studies showed that both CdtB and LtxA (unexpectedly) exerted significant effects on CD4^+^ T cells. As a result we decided to compare the effects of these two prominent *Aa* virulence factors on bone loss using our rat model of *Aa*-induced periodontitis. In this model, *Aa* strains, mutant in *cdtB* and *ltxA*, were compared to their parent non-mutant strains and evaluated for colonization, antibody response to *Aa*, bone loss and disease. We found that bone loss/disease caused by the *ltxA* mutant strain, in which *cdtB* was expressed, was significantly less (p<0.05) than that due to the wild type strain. On the other hand, the disease caused by *cdtB* mutant strain, in which *ltxA* was expressed, was not significantly different from the wild type strain. This data indicates that *Aa* LtxA exerts a greater effect on bone loss than Cdt in this rat model of periodontal disease and supports the utility of this model to dissect specific virulence factors as they relate to immunopathology in studies of *Aa*-induced disease.

## Introduction

*Aggregatibacter actinomycetemcomitans (A. actinomycetemcomitans*) is a Gram-negative coccobacillus that causes localized aggressive periodontitis (LAP) [1]. Localized aggressive periodontitis (LAP) causes rapid loss of ligamentous tissue and alveolar bone surrounding first molars and central incisors, resulting in eventual tooth loss. In the general adolescent population in the U.S., LAP has a prevalence of approximately 0.5%; it is 15 times more prevalent among African-American adolescents, compared to the general population [1]. *A. actinomycetemcomitans* (*Aa*) is also associated with infections of the heart [2], [3], urinary tract [4], and brain [5].

The two most studied toxins of *Aa* are Cytolethal Distending Toxin (Cdt) and Leukotoxin (LtxA) [6], [7]. Cdt targets a multitude of cells and has been shown to damage gingival tissue in rat and human gingival tissue explants [8]. On a functional level Cdt causes cell cycle arrest in rat gingiva when it is applied *in vivo* [9] and induces apoptosis in non-proliferating macrophages [10]. With respect to periodontal disease, Cdt has been linked to induction of RANKL expression in human gingival fibroblasts and periodontal ligament cells [11] and T cells [12]. RANKL interacts with RANK on the surface of osteoclast progenitor cells. This interaction results in the activation of osteoclast progenitor cells to become osteoclasts which resorb bone [13].

Leukotoxin (LtxA), a member of the RTX toxin family [14], [15], [16], is related to the *E. coli* hemolysin alpha [17], [18]. LxtA is expressed as an operon of four genes, *ltxA*, *ltxB, ltxC,* and *ltxD*. The structural gene responsible for cell lysis is *ltxA* *[14]*, *[18]*. The cell receptor for LtxA is human lymphocyte function-associated antigen (LFA-1) [19]. Leukotoxin disrupts the membranes of target cells. It is thought to be active against polymorphonuclear leukocytes and monocytes in humans and Old World monkeys exclusively [20], [21]. Different *Aa* strains produce different amounts of leukotoxin, although a correlation between levels of leukotoxin and disease has not been reported. Overall, leukotoxin is thought to be the major virulence factor of *Aa*-induced aggressive periodontitis as defined by bone loss.

We recently investigated the effect of *Aa* on the rat acquired immune response [22]. Since Cdt is known to affect lymphocytes, and has been shown to affect rat tissue, we also wanted test the effect of cytolethal distending toxin on rat CD4^+^ T cells *in vitro* prior to our testing of a *cdtB* deletion in our rat model of periodontal bone loss. We used LtxA as a control in these lymphocyte *in vitro* studies since previous reports showed that LtxA only affected the lymphocytes of humans and Old World monkeys [21]. Our results showed that leukotoxin and cytolethal distending toxin can affect rat lymphocytes *in vitro.* As a result of these findings we revised our goal to now include *in vivo* studies of the effects of both Cdt and LtxA in a rat model of periodontal disease. In these studies, we are the first to demonstrate that leukotoxin is effective in a rat model of periodontitis and that leukotoxin shows a greater effect on alveolar bone loss when compared to Cdt. Our studies suggest that *Aa-*induced periodontal disease in a rat model can be used to study the interaction of key *Aa* virulence factors and immune regulation on a cellular level.

## Materials and Methods

### Ethics Statement

The animal protocol described in this study was approved by the UMDNJ-Newark Institutional Animal Care and Use Committee protocol number 03024.

### *In vitro* Tests of Cdt and LtxA

#### CdtB protein isolation

We used CdtB as a surrogate marker for the Cdt holoenzyme. CdtB can act alone in *in vitro* tests although it is not as potent as the holoenzyme containing CdtA, CdtB and CdtC, [23].

CdtB protein isolated from IDH781 was cloned into the protein expression vector PET100/DTOPO (Invitrogen). The primers were constructed according to the manufacturer’s recommendation with a CACC at the 5′ terminal end of the forward primer and stop codon added at the 3′ end of the reverse primer end. The *cdtB* gene was amplified by PCR using the following primers:

CdtBF 5′ CACCATGCAATGGGTAAAGCAATT (1351–1370 of the Sugai sequence AB011405.1 (Genbank Accession # AB01140405) and CdtBR2 5′TTAGCGATCATGAACAAAACTAAC (2202–2178 of the Sugai sequence [24]). The cloned *cdtB* gene was verified by sequence analysis using the cloning primers on the plasmid construct. Plasmid DNA of the correct plasmid construct was transformed into the E coli expression strain BL21 (Invitrogen). Induction of *cdtB* expression was performed by incubation with 0.1 mM IPTG after 4 hours of growth. Cells were pelleted and the pellet lysate was run on a (4–20%) pre-cast polyacryamide gel (Bio-Rad), which showed an induced band at approximately around 30 kDa. The gel was blotted and hybridized with anti-poly-His antibody linked to alkaline phosphatase (Sigma), which demonstrated that the cloned band and the induced band were the same. The strain was grown and the cell pellet was lysed under denaturing conditions and the CdtB polyhistidine fused protein was isolated using a Nickel column (Qiagen). The protein isolated was then dialyzed under stepwise dilutions, decreasing the urea concentration to zero, and increasing the pH to pH7.0 to allow the protein to refold. The final buffer condition was 100 mM NaH_2_PO_4_, 10 mM Tris-Cl, pH 7.0. The recombinant protein was tested against the rat lymphocytes.

### *In vitro* Tests: LtxA Protein Source

The leukotoxin protein was provided by Dr. Scott Kachlany (Department of Oral Biology, New Jersey Dental School). The LtxA was isolated as previously described [25]. Briefly, Leukotoxin was isolated from the supernatant of 12 hour cultures of an Aa smooth strain JP2 grown at pH 6.5–7.0. The cultures were centrifuged to remove cells from the supernatant and put through a.22 micron pore size filter to remove any remaining cells. The filtered supernatant was then put through a standard protein concentrator to further purify and concentrate the protein for use in testing against rat lymphocytes.

### *In vitro* Tests: Examination of the Biological Activity of 5–50 ng Doses of CdtB and LtxA on Proliferation of CD4^+^ T cells

CD4^+^ T cells were positively isolated from rats using immunomagnetic bead labeled anti-rat CD4 antibody (clone OX-38) obtained from Miltenyi Biotec. The purified responder CD4^+^ T cells were labeled with 1 µM CFSE (carboxyfluorescein diacetate succinimidyl ester) and cultured in RPMI1640 medium, containing 5% FBS, for 2–5 days at 37°C in 5% CO_2_ incubator. Responder CD4^+^T cells were incubated with graded doses of CdtB or LtxA (5 to 50 ng/ml). Control medium in which CdtB and LtxA were diluted were used, respectively, for the control cells. At the end of the culture periods, flow cytometry was conducted. To reduce non-specific staining in the FACS (fluorescence-activated cell sorter) analyses, a lymphocyte gate on viable cells was imposed, followed by a gate on CFSE^+^ cells, prior to analyses. The extent of CFSE dilution (indicative of proliferation) was determined by computing the mean fluorescence intensity (MFI) of the analyzed cells. FlowJo software (Tree Star) was used to analyze the FACS data.

### Tests of *cdtB* and *ltxA* Mutants in a Rat Model of Periodontal Disease

#### Protocol of our rat model of periodontal disease

In general we followed the rat model protocol we ran in previous studies [26], [27]. We conducted one study using the cdtB mutant strain and a second study using the ltxA mutant strain. Eighteen specific-pathogen-free male Sprague Dawley rats (5–10 week of age) were housed in separate cages and fed powdered food (Laboratory Rodent Meal Diet 5001, Purina Mills Feeds) to promote plaque development. The rats were given 20 mg/ml kanamycin and 20 mg/ml ampicillin in their drinking water for four days to depress the resident flora. During the final two days of antibiotic treatment, the rats’ mouths were also swabbed with chlorhexidine gluconate 0.12% rinse. Following a rinse-out period of three days the rats were divided into three groups of six rats per group for each study. In the first set of experiments, Group 1 was the control and received no bacteria. Group 2 received the cdtB mutant HS1031Rif. Group 3 received wild type Aa strain IDH781NRif. The bacteria inoculation was given three hours after fasting. The inoculum given to Group 2 or Group 3 consisted of 108 bacterial cells in 3% sucrose PBS mixed with a small amount of powdered food, put in special feeder trays, which fit over the bedding in the cage. The Aa strains were grown and inocula were prepared as previously described [27]. After an hour the inoculated food was removed and replaced with regular powdered food. This inoculation-feeding regimen was repeated for eight days. The rats were then fed powdered food for a month. The rats were then switched to pellet food to prevent overgrowth of the incisor teeth. In the second study, Group 1 was the control, Group 2 was fed the ltxA mutant strain Aa1700Rif and Group 3 received the wild type strain DF2200Rif.

Two weeks after bacterial inoculation, the rats’ oral flora was sampled with a cotton tip swab for soft tissue and a Stimudent (Johnson & Johnson) for hard tissue. Dilutions of the samples were plated on enriched trypticase soy agar (ETSA)+10%Sheep Blood at 37°C for three days for total counts and on AaGMRif35 plates at 37° with 10% CO_2_ for three days to select for the introduced *Aa*.

Twelve weeks past the final bacterial feeding, the oral flora of the rats was again sampled. The rats were euthanized by intraperitoneal injection of sodium pentobarbital (100 mg/kg), at which time blood samples were taken by cardiac puncture to determine post-inoculation relative *Aa* antibody levels, and the upper jaws were removed for the assessment of bone loss.

#### Assay for Aa colonization

The oral flora of the rats was sampled as described above and plated on ETSA +10% sheep blood for total bacterial counts and on *Aa*GMRif35 plates to select for *Aa*. The colonies were counted on both types of plates and expressed as CFU (colony forming units) per ml of collected sample in PBS. For each oral sample, the CFU/ml for *Aa* plates was divided by the total bacteria CFU/ml for the sample to correct for variation in sampling. The means of the ratios of Aa/total CFU/ml were calculated for each rat treatment group and the means were compared by ANOVA analysis. The first and second studies were handled in identical manner.

#### Assay for bone loss and disease

Our methods for measuring bone loss and disease were previously described [26] and used in this study. Photos of the stained jaws and teeth were taken using a DP12 microscope digital camera system (Olympus) at a 9.2× magnification. The bone loss was measured in a blind manner in duplicate by an examiner. The vertical distance between the cemento enamel juction (CEJ) and the alveolar bone crest (ABC) was measured at 10 sites on the three maxillary molars on each sides of the jaw [26] [22].

#### Bone loss and disease determination

The raw bone loss data for each group was decoded and sent to an independent statistician. The mean and standard deviation of total bone loss at each site was calculated for each of the treatment groups. A diseased site was defined as a site where the measurement of bone loss (defined as the distance between the cemento-enamel junction and the bone crest) was greater than two standard deviations above the mean of the bone loss measure for that site in the control group. If a rat had two or more diseased sites per jaw, the rat was considered a diseased rat. To compare the associations between groups and disease, the groups were compared using Fisher’s Exact Test and the Cochran Armitage Trend Test. The results were considered significant at a probability level of p<0.05. The results of the determination of diseased rats between the two reads were compared using a measure of agreement (kappa) score [26].

#### Antibody level to *Aa*

Relative *Aa* IgG antibody levels was determined by ELISA, as previously described [22]. Preinoculation tail bleeds were taken from two rats in each experimental group. Blood was also collected from all rats by cardiac puncture after their sacrifice at 12 weeks after inoculation. To identify the level of background reaction to the *Aa*, sera from preinoculated rats and sera from the control group of uninoculated rats were also tested for their reaction to *Aa.*

#### Construction of *cdtB* mutant

The cloning vector and the vector used for transformation into Aa was PVK50. This plasmid was constructed by first amplifying a region of Aa containing two copies of the Aa uptake sequence [28], using primers USS-F and USS-R which amplified a 210 bp region between sodA and and gene AA00069 in the HK1651genome (http:/www.oralgen.lanl.gov/coordinates 52,230–52,341). The template strain DNA was from HK1651. The primers were designed to add a HindIII site at one of the fragment and a KpnI site at the other. The 220 bp PCR product was digested with HindIII and KpnI and ligated into the HindIII/KpnI sites of LITMUS28 (New England Biolabs).

Portions of the *cdtB* and *CdtC genes* of Aa strain DF2200N were amplified by PCR (using primers CC4 5′AGGTTAAACACAGTATGA3′ bases 2569–2545 of sequence reported by Sugai (GenBank accession # AB01140405.1) [24] and CB6 5′ACAGTGCATGCTTGGCCA3′ bases 1822–1831 of Sugai’s sequence(GenBank accession # AB01140405.1) to generate a 747 bp band. For the PCR reaction, Ready-To-Go PCR beads (GE Healthcare) were used according to the manufacturer’s instructions. The PCR reaction product was purified by using a Qiaquick PCR purification kit (Qiagen).

The amplified fragment was then treated with *DNA polymerase*I large fragment, Klenow Enzyme (Roche), to blunt the ends of the fragment. The vector pVK50 was digested with restriction enzyme *EcoR*V (Invitrogen) according to manufacturer’s instruction, to generate a blunt ended cut in the plasmid. The cut plasmid and the *Aa* PCR fragment was then ligated using T4 ligase (Roche). The ligation reaction (2 µl) was used to transform One ShotTOP10F’ *E. coli* competent cells (Invitrogen) and the resulting ampicillin resistant colonies were grown on LBamp50 plates. Clones with inserts were identified by agarose gel electrophoresis. The cloned 747 bp PCR fragment was verified by sequencing the insert using the CC4, CB6 primers. This plasmid was named HS65. The plasmid was mutagenized by Tn5 insertions *in vitro* using the EZ-Tn5<KAN-2> Insertion Kit following manufacturers’ instructions (Epicenter). When *E. coli* TOP10 was transformed with the mutated plasmid, colonies containing the transposons were resistant to kanamycin 50 µg per ml in agar plates. Transposon insertions in *cdtB* were identified by amplifying the *cdtB* gene using primers CB-1 (5′ ATGCAATGGGTAAAGCAATTA bases 1351–1372 of the Sugai sequence [24]) and CB-2 (5′TTAGCGATCATGAACAAAACTAAC bases 2202–2178 [24]). The location of the transposon was found by sequencing from the ends of the cloned fragment using primersCC4 and CB6. The transposon was inserted at base 2102 of the sequence reported by Sugai [24], in codon 252 of *cdtB*. Plasmid DNA from the clone containing the *cdtB* insertion was isolated using Qiaprep spin miniprep kit (Qiagen). This plasmid was named HS66. *Aa* strain IDH781Nal (pMB7) [29] was transformed with HS66 DNA using an inducible *tfox* system [29]. The position of the transposon in the bacterial strain was verified by sequencing, analysis using primers CC4 and CB6. The IDH781Nal *Cdt B* mutant was named HS1030. For use in the animal model we selected a rifampicin resistant mutant clone of HS1030 which was able to grow on AaGMRif35 plates. This IDH781NRif strain with the *cdtB* transposon mutation was called HS1031. Plasmids and strains are described in Table 1.

**Table 1:** Strains and Plamids used in this Study.

Strain or Plasmid	Relevent characteristic	Reference or Source
**Plasmids**		
Litmus 28	Cloning vector Amp^R^	New England Biolabs
PVK50	Litmus28 with *A. actinomycetemcomitans* uptake sequence, Amp^R^	Dr. Kablilan VelliyagounderThis work
pHS 65	pVK50 with parts of *cdtA* and *cdtB,* Amp^R^	This work
pHS 66	pHS65*cdtB*::tn5<KAN -2> Amp^R^ Kam^R^	This work
PET100/D-TOPO	Protein cloning and expression vector, Amp^R^	Invitrogen
Aa1700	LtxA mutant of NJ2200	[30]
***A. actinomycetemcomitans*** ** Strains**		
IDH781nal	*A. actinomycetemcomitans* clinical isolate, serotype d Nal^R^	[29]
HS1030	IDH781nal *cdtB*::tn5<KAN -2> Kam^R^ Nal^R^	This work
HS1031	HS1030 spontaneous Rif^R^ mutant	This work
DF2200rif	NJ2200 spontaneous Rif^R^	[38]

#### *ltxA* mutant

The *ltxA* mutant strain Aa1700 and its parental unmutated strain DF2200 were provided by Dr. Scott Kachlany (Department of Oral Biology NJ Dental School). The *LtxA* mutant’s construction was previously described [30] using the method developed by M.K. Bhattacharjee [29]. The general details of this method used was the same as that used for the CdtB mutant, except the transposon insertion was in the *ltxA* gene and the parental strain was DF2200. Rif resistant strains were selected by plating lawns of bacteria on Rif35 plates and selecting for Rif resistant colonies.

#### Statistical analyses

All analyses were done using JMP software (SAS). For analyses of the effect of toxins on lymphocytes the MFI of the test lymphocyte cultures were compared with control cultures (0 ng/ml CdtB or 0 ng/ml LtxA) using two-tailed Student’s t-test (p<0.05). Significance of *Aa* infection was assessed by comparing the total *Aa* CFU/total bacterial counts per group by ANOVA analysis. *Aa*-induced antibody production was determined by comparing serum antibody response in *Aa*-fed rats to that of control rats using ANOVA and Tukey’s post hoc test. Student’s t-test was used, where appropriate, to determine statistical significance (p<0.05). For determination of significance of bone loss the means of the total bone loss per group were compared by ANOVA analysis. Comparison of associations between groups and disease, were determined using Fisher’s Exact Test and Cochran Armitrage Trend test.

## Results

### Effect of CdtB and LtxA on Proliferation of CD4^+^ T cells

**Figure 1:**
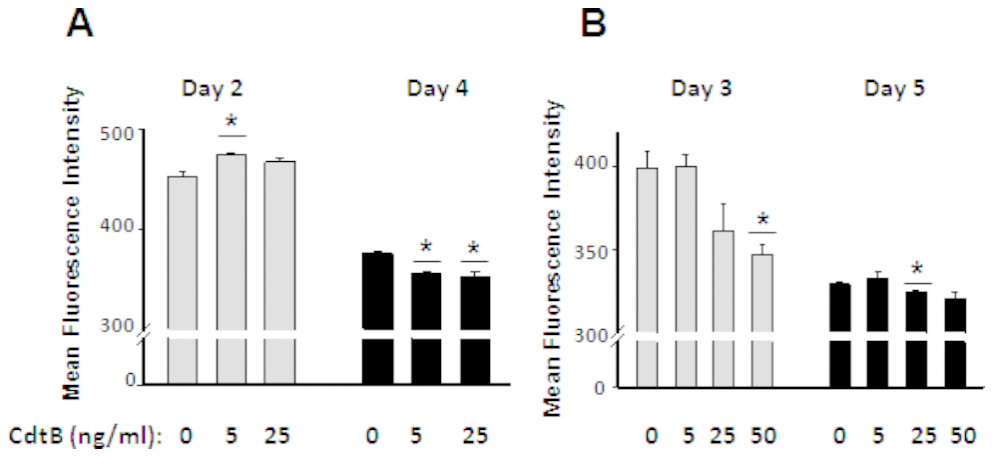
Proliferation of rat CD4^+^ T cells in response to CdtB in vitro. Values represent mean fluorescence intensity (MFI) of triplicate cultures of CFSE-labeled rat CD4^+^ T cells. (A) Cultures were terminated at days 2 and 4 (experiment 1). (B) Cultures were terminated at days 3 and 5 (experiment 2). The data are represented as means ± SEM. Increase in MFI of CFSE staining indicates inhibition of proliferation. Statistics were calculated by two-tailed t-test; *P<0.05, in comparisons to control cultures (0 ng/ml CdtB).

The effect of CdtB on proliferation of rat CD4^+^T cells appears to depend on the dose and duration of culture (Fig. 1). Early in the response (day 2), CdtB significantly (p<0.05) inhibited the proliferation of CD4^+^ T at 5 ng/ml (Fig. 1A). However, by day 4 of culture 5 and 25 ng/ml, CdtB induced significant (p<0.05) proliferation (decrease in CFSE MFI) of CD4^+^ T cells, (Fig. 1A). By day 3 of culture, 50 ng/ml of CdtB induced proliferation of CD4^+^ T cells in experiment 2 (Fig. 1B). Similarly, by day 5 of culture, 25 ng/ml CdtB induced significant (p<0.05) proliferation of CD4^+^ T cells. (Fig. 1B).

**Figure 2:**
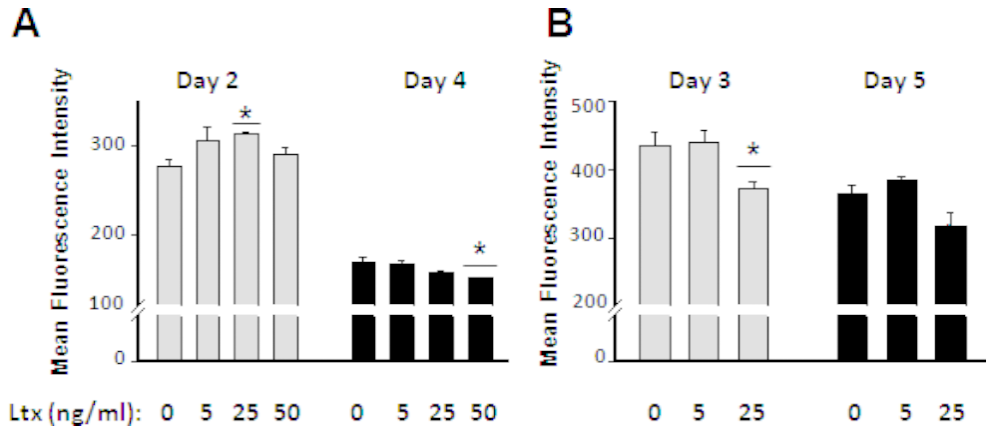
Proliferation of rat CD4^+^T cells in response to LtxA in vitro. Values represent mean fluorescence intensity (MFI) of triplicate cultures of CFSE-labeled rat CD4^+^ T cells. (A) Cultures were terminated at days 2 and 4 (experiment 1). (B) Cultures were terminated at days 3 and 5 (experiment 2). The data are represented as means ± SEM. Increase in MFI of CFSE staining indicates inhibition of proliferation. Statistics were calculated by two-tailed t-test; *P<0.05, in comparisons to control cultures (0 ng/ml LtxA).

LtxA was added to the cells originally to act as a negative control based on previous studies that found that LtxA was active only on the white blood cells of Humans and Old world monkeys [21]. Unexpectedly, LtxA had an effect on the rat CDT4^+^ T cells. Examination of LtxA for its proliferation effects on rat CD4^+^ T cells revealed that, similar to CdtB, LtxA significantly (p<0.05) inhibited the proliferation of rat CD4^+^ T cells at an early point (2 days) of culture, at 25 ng/ml (Fig. 2A). However, at later time points LtxA significantly (p<0.05) induced proliferation of rat CD4^+^ T cells, at 50 ng/ml and 25 ng/ml in experiments 1 and 2 (Fig. 2A and 2B, respectively).

It does seem that both CdtB and LtxA have similarities in their effects on proliferation of rat CD4^+^ T cells at early (day 2) and late (days 3–5) time points.

We also measured the viability of the CD4^+^ T cells responding to LtxA at day 4. There was no significant difference in viability of the cells due to LtxA. Viabilities for 0, 5, 25, and 50 ng/ml LtxA were 82.5, 90.7, 84.2, and 77.4%, respectively (a slight decrease at 50 ng/ml).

### Rat Model of Periodontal Disease

#### Colonization

Colonization by the *cdtB* mutant strain was comparable to the wild-type strain (Table 2). The CFU of *cdtB* mutant strain devided by total CFU was a mean of 21.01±26×10^−4^ at 2 wks and 0.68±.09×10^−4^ at 12 weeks. The wild type strain group divided by total flora CFU was a mean of 16.36±15×10^−4^ at 2 weeks and 7.16±9.6×10^−4^ at 12 weeks. At both 2 and 12 weeks, the Aa/total count means were not significantly different between Group 2 (the *cdtB* mutant) and Group 3 (IDH781 wild type). The means of the *Aa* counts/total bacterial count per group dropped from the 2 week sampling to the 12 week sampling for both *cdtB* plus and *cdtB* minus groups.

**Table 2:** Incidence of disease, bone loss and extent of *Aa* colonization in *cdtB* mutant and control rats.

	Disease	Bone Loss	*Aa* counts/total counts×10-4	
			2 wks	12 wks
**Control**				
1–1	no	121.41	0.000	0.000
1–2	no	126.40	0.000	0.000
1–3	no	140.33	0.000	0.000
1–4	no	112.92	0.000	0.000
1–5	no	113.98	0.000	0.000
1–6	no	135.60	0.000	0.000
**avr±SD**		**125.11±11.2**	0.000	0.000
***cdtB*** ** mutant**				
2–1	yes	136.00	0.000	0.000
2–2	yes	137.56	60.000	2.270
2–3	no	121.29	10.300	0.520
2–4	no	121.35	6.800	0.000
2–5	yes	147.32	49.000	1.140
2–6	no	122.86	0.000	0.130
**avr±SD**		**131.07±10.8**	**21.01±26**	**0.68±.09**
**WT 1DH781**				
3–1	yes	139.74	2.360	9.040
3–2	yes	152.60	37.590	0.200
3–3	yes	166.70	20.950	24.580
3–4	yes	139.34	0.000	0.000
3–5	yes	135.85	10.340	0.000
3–6	no	130.75	26.920	9.150
**avr±SD**	†	**144.16±13.2** *	**16.36±15**	**7.16±9.6**

* WT compared to Control is significantly different p<0.05 by ANOVA and Tukey's Studentized Range post-hoc test.

† WT and *cdtB* mutant are significantly different from Control by Fisher's Exact Test and.

Cochran Armitage Trend Test.

Colonization by the wild-type and mutant strains of *ltxA* are described in Table 3. The *ltxA* mutant strain group divided by total flora CFU was a mean of 16.31±31.6×10^−4^ at 2 wks and 0.49±.072×10^−4^ at 12 weeks The wild type strain group divided total flora CFU was a mean of 33.61±81.0×10^−4^ at 2 weeks and 0.36±0.69×10^−4^ at 12 weeks. Colonization by the *ltxA* positive stain and the *ltxA* negative strain was not significantly different at 2 weeks and 12 weeks. Again, the means of the *Aa* counts/total bacterial counts dropped from 2 weeks to 12 weeks.

**Table 3:** Incidence of disease, bone loss and extent of *Aa* colonization in *ltxA* mutant and control rats.

	Diseased	Bone Loss	*Aa* counts/total counts ×10-4	
			2 wks	12 wks
**Control**				
1–1	no	72.87	0.000	0.000
1–2	no	97.05	0.000	0.000
1–3	no	105.65	0.000	0.000
1–4	no	125.40	0.000	0.000
1–5	no	90.93	0.000	0.000
**avr±SD**		**98.377±19**	0.000	0.000
***ltxA*** ** mutant**				
2–2	no	100.86	0.000	0.000
2–3	no	109.79	80.300	0.310
2–4	no	105.01	3.300	0.230
2–5	no	98.57	0.580	0.000
2–6	no	105.70	11.190	1.920
2–7	yes	118.59	2.500	0.490
**avr±SD**		**106.416±7.1**	**16.31±31.6**	**0.49±0.72**
**DF2200 (wt)**				
3–1	yes	116.72	0.160	0.230
3–2	yes	128.20	199.000	0.200
3–3	yes	115.26	0.000	0.000
3–4	no	117.61	0.000	0.000
3–5	yes	130.07	1.100	0.000
3–6	no	106.24	1.390	1.770
**avr±SD**	†	**119.014±8.1** *	**33.61±81.0**	**0.36±0.69**

* WT compared to Control or *ltxA* mutant is significantly different p<0.05 by ANOVA and Tukey's Studentized Range post-hoc test.

† WT is significantly different from Control or *ltxA* mutant by Fisher's Exact Test and Cochran Armitage Trend test.

Some of the data shown in Table 3 appeared in one of our previously published manuscripts [26]. The data are in Tables 2 and 4 of that manuscript.

#### Total bone loss and treatment group

For Cdt (Table 2), the mean total bone loss seen in IDH781 (wild type strain) was significantly different from that of the negative control by ANOVA and Tukey’s studentized range post–hoc test. The *cdtB* mutant group, had more mean total bone loss than the negative control group and less than the wild type group; but the differences were not significant.

For LtxA (Table 3), using ANOVA and Tukey’s studentized range post–hoc test, the mean total bone loss of the wild-type group was significantly different from the mean of the control group and the mean of the *ltxA* mutant group. In all cases the wild type group showed more bone loss than the *ltxA* mutant group and the control group. All differences were significant (p<0.05).

#### Disease and toxin group

For Cdt (Table 2), the results of Fischer’s Exact and Cochran Armitage Trend test showed that there was a significant difference in frequency of disease between wild type IDH781 treatment (5/6 diseased), and the control group (0/6 diseased) p<0.05) The highest level of disease was found in the wild type IDH781 group, while the lowest was found in the uninoculated control group. Again using Fischer’s exact test and the Cochran Armitage Trend test the *cdtB* mutant Group, had significantly more disease than the control group (mutant vs control = 3/6 diseased vs 0/6 diseased ) p<0.05, however the mutant strain was not significantly different from the wildltype group (mutant strain vs wildtype = 3/6 diseased vs 5/6 diseased). The mutant strain was significantly different from the control strain (mutant 3/6 diseased vs control strain 0/6 diseased.

For LtxA (Table 3), using Fisher’s exact test and Cochran Armitage Trend test there was a significant difference between the wild type group DF2200 (4/6 diseased) and the uninoculated control group (0/6 diseased). There was also a significant difference between the wildtype group (4/6 diseased) and the *ltxA* mutant group (1/6 diseased). The control group (0/6 diseased) was not significantly different from the *ltxA* mutant group (1/6 diseased). However, in this case the *ltxA* the mutant strain was significantly different from the wildtype strain (wildtype 4/6 diseased vs mutant 1/6 diseased).

Based on these results, the *ltxA* mutant strain has a greater effect on bone loss than does the *cdtB* mutant because it shows significantly less bone loss than the wildtype strain. While the *cdtB* mutant strain shows a trend in the direction of bone loss, the data does not achieve significance. Put another way, the disease caused by *cdtB* mutant strain, in which *ltxA* is expressed, is not significantly different from the wild type strain with respect to the bone loss and disease in this experimental model. This data suggests that LtxA shows more of an association with disease than seen with Cdt.

#### Antibody to *Aa*

In both the of the *cdtB* mutant and the *ltxA* mutant experiments, the level of antibody to *Aa* was higher in the *Aa*-inoculated groups compared to the preimmune sera. However, in neither case were antibody levels in the inoculated strains significantly different from the uninoculated strains; although the antibody levels were higher.

## Discussion/Conclusions

This study was designed initially to examine Cdt, a key *Aa* virulence factor, using a rat model of *Aa*-induced periodontal disease. We knocked-out the *Aa cdtB* gene to assess its effect on bone loss, assuming that the *cdtB* mutant strain would show reduced bone loss as compared to the parent (control) *Aa* strain that expressed Cdt. Our initial thought was that an *Aa ltxA* knockout would serve as a second control since Cdt was still expressed and bone loss would be unaffected. This design was based on the fact that LtxA, was previously thought to be active only in humans and Old world monkeys [20], [21]. However in our *in* *vitro* study LtxA showed significant activity with rat lymphocytes. Moreover, in a recent study, LtxA has been shown to affect viability of rat lymphocytes *in vivo* [31]. In line with the studies referenced above, results from the current study showed that LtxA was effective in rats *in vivo* and suggest that both virulence factors (Cdt and LtxA) can be studied in a time dependent manner using this rat model of periodontitis. With that as a foundation, this model should be able to contribute to our understanding of *Aa*-induced human periodontal pathogenesis.

It is clear that all animal models have some limitations; however, when well designed they provide information that extends far beyond what can be learned from *in* *vitro* studies, which are typically limited to specific cellular response to one cell type [32]. In contrast, an animal model can uncover complex cellular interactions [32]. In the experiments we report herein we used our *in vitro* system to guide the animal experiments. Our initial expectation was that leukotoxin would be ineffective in our rat model; however our results *in vitro* proved contrary to that assumption. As a result we decided to test leukotoxin in our animal model of *Aa*-induced bone loss. Our methods of assessing bone loss using this model appears to be very sensitive since the model was able to differentiate between LtxA and Cdt induced disease.

The *in vitro* studies showed that depending on the dose and duration of the toxin under culture conditions, CdtB and LtxA could either inhibit or induce proliferation of rat CD4^+^ T cells. The basis of this dichotomy in the response of CD4^+^ T cells to CdtB is not entirely clear at present. However, the early inhibition of proliferation of CD4^+^ T cells that we have observed, is likely a manifestation of cell cycle arrest in either the G_1_/S or G_2_/M transition phases, and or apoptosis induction, as observed for Cdt toxins [33]. Indeed, CdtB was detectable in nuclei of CHO-K1 cells by 4.5 h post intoxication; and, clear evidence of cell membrane damage was seen as early as 18 h post intoxication of human CD4^+^ T cells; and cell cycle arrest, in response to CdtB, is both dose and time dependent [34]. However, it is entirely possible that the dichotomous effects of the toxins relate to phases of the adaptive immune response to the toxins. We had previously shown that intact *Aa* induces a significant effect on the adaptive immune response that affects both B and CD4^+^ T cells [22]. It is also reasonable to speculate that exposure of lymphocytes to LtxA could induce a range of biological signaling cascades that could initially either directly or indirectly (via soluble factors), and thus inhibit lymphocyte proliferation. Similarly, at later time points, soluble factors produced could induce proliferation in lymphocytes. Depending on the factor(s) produced, the effect at later time points could be varied. Irrespective of the effects observed in our studies, it is certain that LtxA exhibits effects on CD4^+^ T cells. It has been shown using the adoptive transfer of Aa antigen specific T lymphocytes that T cell specific bone loss can be induced via osteoclastic activation [35]. Further, T-cell-derived RANKL expression can be blocked with kaliotoxin which also inhibited antigen stimulated Th1 cell proliferation and T cell RANKL expression [36]. Indeed in our previous study [22] we observed upregulation of RANKL in lymphocytes in our rat feeding model where we observed bone loss in disease induced by a wild type *Aa* strain. In that study upregulation of a series of bone morphogenic proteins such as BMPs 2, 3, 10, and 11 were observed suggesting *Aa-*induced regulation of bone loss in our rat model. Overall, those studies suggest that *Aa* affects T cells, which affect bone loss through RANKL expression. Now that we are aware of the activity of leukotoxin in our rat model we plan to examine this relationship in a systematic fashion to determine the effect of leukotoxin on periodontal pathogenesis.

In future studies, we will investigate the expression of cytokines from T cells in a time dependent manner, comparing the effects of a *cdtB* mutant versus a *ltxA* mutant. We would expect to collect rat lymphocytes at various time points during the infection [22], and then assess the presence and amounts of various cytokines derived from these *Aa* exposed lymphocytes.

With respect to *Aa* colonization, 19 of the 24 rats inoculated with *Aa* showed detectable *Aa* by culture and 15 of 24 rats showed detectable *Aa* 12 weeks after infection. In most cases 12 week samples had reduced percent of *Aa* relative to the total flora. None of the 12 uninoculated control rats showed any cultivable *Aa.* It is possible that *Aa* would have been detected in all rats had we used PCR, which we have seen in previous studies [22]. However, because our goal was to study the effects of LtxA and Cdt we thought it best to assess viable *Aa* as opposed to DNA. *Aa* was presented as a fraction of the total flora which varied from 0.36±0.69×10^−4^ to 7.16±9.6×10^−4^ at 12 weeks in mutant strains. The amounts of Aa as a percentage of total flora when collecting subgingival flora from diseased sites in human cases of LAP also vary widely. In this study plaque samples were collected from a full arch while disease occurred in one or two teeth. Since most of the teeth sampled did not have disease, it is possible that the low percentage of *Aa* is due to its dilution by commensal members of the flora. One survey in humans showed the variation of *Aa* was 0.2%–5.0% of total flora; while a second survey showed variation from 0.1% to 114% [37]. Variation could be due to sampling and recovery but in general these numbers should be considered as estimates.

One weakness in this study was the fact that no significant differences in the relative antibody response to *Aa* in both rat experiments were seen at 12 weeks post inoculation. A possible explanation for this finding could be the fact that active disease occurred at an earlier stage and that at 12 weeks disease was in remission. In a recent study [22] we found that the antibody level peaked 2–4 weeks after inoculation. Therefore, had we taken the samples earlier we might have shown significant differences in the various rat groups. In addition, differences may have been seen if we increased the numbers of animals in the treatment groups. This same explanation could be given for the effect of Cdt on bone loss. Our statistical analysis suggests that increasing the numbers of infected rats to 12 could achieve significance with respect to Cdt.

In conclusion, we have shown that LtxA (and to some extent Cdt) are involved with bone loss and disease in our rat model of periodontal disease. Our *in vitro* studies demonstrate that, as observed for intact *Aa*, toxins elaborated by *Aa* (including LtxA and Cdt) exhibit immune-modulating effects on rat CD4^+^ T cells. Previous studies have shown that infection of rats with wild type *Aa in* *vivo* resulted in induction in of an array of cytokines and bone morphogenic family of proteins in lymphocytes, which may play a role in bone resorption [22]. The extent to which LtxA and Cdt induce immunoregulatory effects that culminate in bone resorption, remain to be explored in future *Aa*-infected rat experiments.
